# A defect in the inner kinetochore protein CENPT causes a new syndrome of severe growth failure

**DOI:** 10.1371/journal.pone.0189324

**Published:** 2017-12-11

**Authors:** Christina Y. Hung, Barbara Volkmar, James D. Baker, Johann W. Bauer, Emanuela Gussoni, Stefan Hainzl, Alfred Klausegger, Jose Lorenzo, Ivana Mihalek, Olaf Rittinger, Mustafa Tekin, Julia E. Dallman, Olaf A. Bodamer

**Affiliations:** 1 Division of Genetics and Genomics, Boston Children’s Hospital, Boston, Massachusetts, United States of America; 2 Harvard Medical School, Boston, Massachusetts, United States of America; 3 Department of Pediatrics, Paracelsus Medical University Salzburg, Salzburg, Austria; 4 Department of Biology, University of Miami, Coral Gables, Florida, United States of America; 5 Department of Dermatology, EB House Austria, University Hospital of the Paracelsus Medical University Salzburg, Salzburg, Austria; 6 John P. Hussman Institute for Human Genomics, University of Miami, Miami, Florida, United States of America; Duke University, UNITED STATES

## Abstract

Primordial growth failure has been linked to defects in the biology of cell division and replication. The complex processes involved in microtubule spindle formation, organization and function have emerged as a dominant patho-mechanism in these conditions. The majority of reported disease genes encode for centrosome and centriole proteins, leaving kinetochore proteins by which the spindle apparatus interacts with the chromosomes largely unaccounted for. We report a novel disease gene encoding the constitutive inner kinetochore member CENPT, which is involved in kinetochore targeting and assembly, resulting in severe growth failure in two siblings of a consanguineous family. We herein present studies on the molecular and cellular mechanisms that explain how genetic mutations in this gene lead to primordial growth failure. In both, affected human cell lines and a zebrafish knock-down model of Cenpt, we observed aberrations in cell division with abnormal accumulation of micronuclei and of nuclei with increased DNA content arising from incomplete and/or irregular chromosomal segregation. Our studies underscore the critical importance of kinetochore function for overall body growth and provide new insight into the cellular mechanisms implicated in the spectrum of these severe growth disorders.

## Introduction

Primary growth failure disorders, including primary microcephaly and primordial dwarfism, are well-documented consequences of cell division defects in human. To date the majority of identified molecular causes center around DNA repair and genome replication, centriole formation and centrosome function, or mitotic spindle and cell cycle regulation [1–4]. Of these, centrosome and centriole proteins have emerged as crucial players in the pathogenesis of severe microcephalic dwarfism during recent years. However, defects in another subcellular network, the kinetochore, have been identified in only few individuals with the same primary growth failure phenotype. Correct localization and assembly of the tri-laminar kinetochore complex (inner centromere, inner kinetochore, outer kinetochore) [5, 6] to the centromere is required for faithful segregation of sister chromatids during cell division. To date, mutations in only two kinetochore members have been associated with human disease: *KNL1* (*CASC5*, MIM:*609173) as cause of primary microcephaly [7] and *CENPE* (MIM:*117143) as cause of profound microcephalic primordial dwarfism [8]. Both proteins are members of the outer kinetochore which links spindle microtubules to the inner kinetochore and subsequently to sister chromatids during cell division. Given that at least 30 protein complexes comprise the kinetochore [9] and the widespread clinical implementation of next-generation sequencing, it appears that defects in kinetochore assembly or function are currently under-represented as cause for primordial dwarfism with primary microcephaly. Here, we report the addition of an inner kinetochore member *CENPT* (MIM:*611510), which is vital for kinetochore assembly, to the repertoire of genes implicated in primordial dwarfism.

## Clinical description

**Fig 1:**
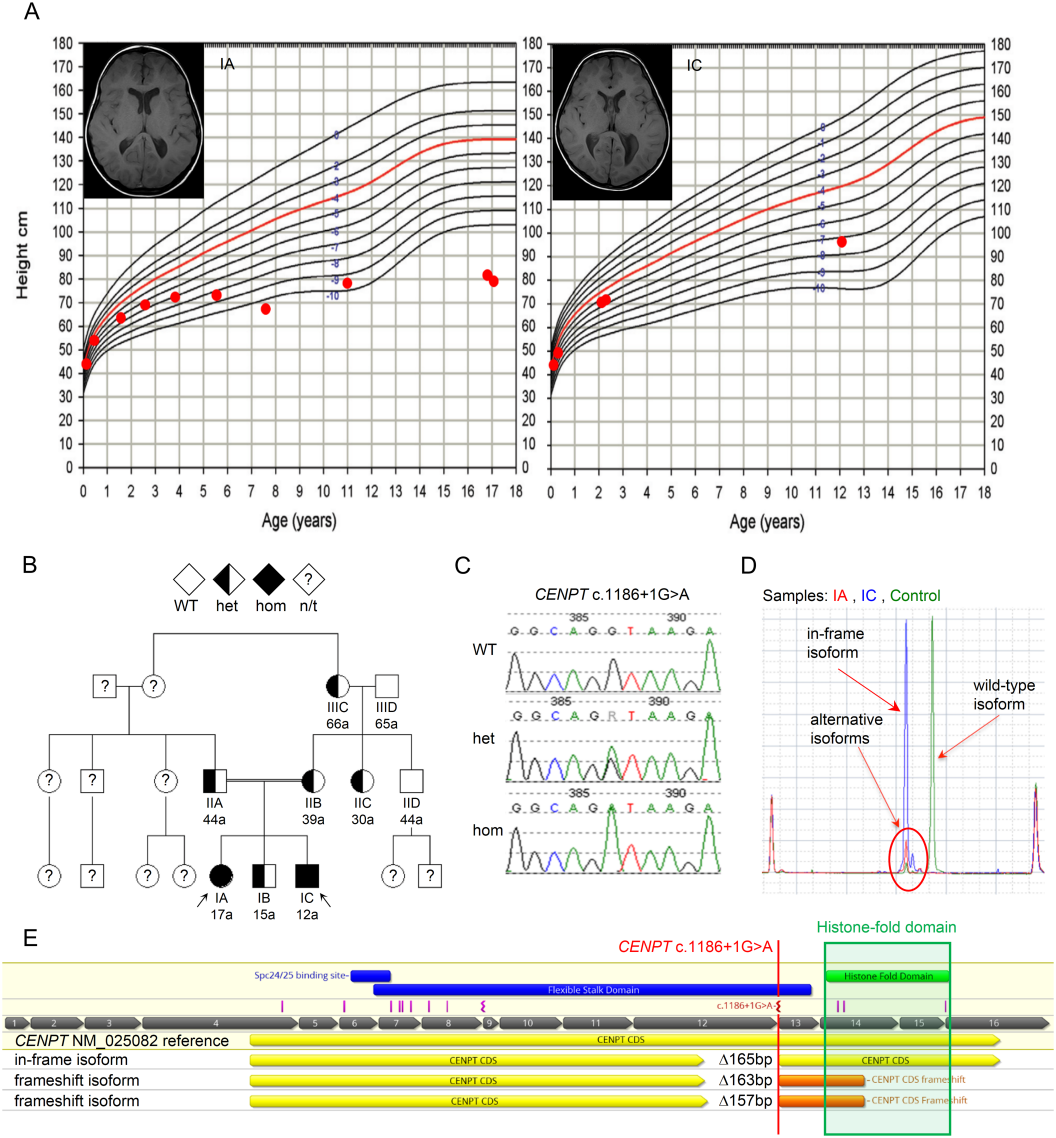
Phenotypic presentation of *CENPT* defect. (A) The left panel shows height measurements on female primordial dwarfism growth curve and MRI of IA. The right panel shows height measurements on male primordial dwarfism growth curve and MRI of IC. (B) Pedigree and (C) Sanger sequence trace. (D) RT-PCR data on a BioAnalyzer chip of index patients (red and blue peaks) and control (green) are shown. The wild-type isoform is only seen in the normal control. Among the alternatively spliced isoforms (red circle) the in-frame isoform (left peak) shows highest expression. This endogenously present isoform is only minimally expressed in the normal control. (E) Schematic of human CENPT. Red marking annotates the splice-mutation in the index cases. Pink markings represent the variants found in 9 individuals of 8 families identified through database searches and clinical contacts. The green box encircles the C-terminal conserved histone-fold domain (HFD). On the bottom, the three alternative isoforms are schematically represented. In all isoforms part of exon 12 is spliced out. The out-of-frame isoforms result in premature termination codons, which obliterate most of the HFD.

The index patient, a 12 year-old girl (IA) at presentation, was born at term as the first child of healthy parents following an uneventful pregnancy. Her birth parameters were documented as follows: birth length of 46cm (3^rd^ centile [10]), birth weight of 2400g (3^rd^ centile [10]) and head circumference (HC) of 31cm (3^rd^ centile [10]). Within a few months of life, all growth parameters were well below the 3^rd^ centile (Fig 1A). APGAR scores were 9/10/10. No peri- or neonatal problems were noted with the exception of gastroesophageal reflux in the first weeks of life. Clinical examination at presentation revealed mild craniofacial dysmorphic features including narrow face, down-slanting palpebral fissures and a beak-like nose. Neurodevelopmental evaluation and skeletal surveys revealed global psychomotor developmental delay and dissociated retardation of bone age. At 6.25 years her carpal bone age was that of a 2 year-old although her epiphyseal bone age was that of a 5 year-old girl. Additional findings include bilateral hyperopia (+9 sph at 17 years) in absence of other eye abnormalities, mild scoliosis, box-shaped lumbar vertebral bones (L2-5) and length discrepancy of the legs (left > right). Growth hormone therapy was administered until approximately 7 years of age without any obvious benefit. At the age of 12 years she was 86.7cm tall (-9 SD [11]), weighed 11.35kg (-6 SD [10]) and had a HC of 46.5cm (-6 SD [11]). At her most recent follow-up at 17 years of age her growth parameters had not changed, representing stagnation in growth since the age of 12 years (Fig 1A). At the age of 17 years she has not undergone puberty despite normal FSH, LH, estradiol and prolactin levels. Except for some pubic hair development (Ph4) no other sexual characteristics (breast, axillary hair, menarche) have developed.

The affected brother (IC) was born at 41 weeks of gestation as the third child with a birth length of 45cm (<3^rd^ centile [10]), birth weight of 3100g (25^th^ centile [10]) and a HC of 34.5cm (25^th^ centile [10]). He had APGAR scores of 5/9/10. A combination of failure to thrive, feeding difficulties and small genitalia prompted a normal diagnostic work-up for Prader-Willi syndrome by fluorescence in situ hybridization (FISH), and normal subtelomeric FISH and karyotype analyses. Physical examination at 6 weeks of age identified additional features including small mouth, downslanting palpebral fissures, abnormal palmar creases and hypoplasia of the fingernails. No muscular hypotonia or major malformations were noted. Mild hyperopia (+1 sph) was diagnosed at 3.5 years of age without need of correction. He underwent bilateral orchidopexy and circumcision at 4 years of age due to inguinal small testes and phimosis. At 12 years of age, he was noted to have an empty scrotum and micropenis for which he was treated with 3 doses of testosterone without notable effect. However, his pubic hair development (Ph2) has started at 12 years of age. His skeletal status shows bilateral genua valga and dissociated bone age similar to his sister’s. At 12 years, his phalangeal bone age is that of a 10 year-old while his carpal and epiphyseal bone age of the upper extremities trail far behind to an age as young as 6 years. His height is 97 cm (-7 SD [11]) (Fig 1A), his weight 19.5 kg (-4 SD [10]) and HC 49.5 cm (-4 SD [11]).

To date no seizures or other neurologic deficits have been reported. Their brain MRIs have shown no structural abnormalities except for overall small brain size (Fig 1A). Their unaffected brother (IB) is of normal stature, weight and intelligence as are both parents (IIA and IIB). No abortions, stillbirths or other affected individuals have been reported in the family.

## Results

Autozygosity mapping following SNP microarray of a consanguineous Turkish family with one unaffected and two affected children (Fig 1B) revealed 5 shared regions of homozygosity, which were larger than 1Mb in size. Candidate gene prioritization using ToppGene [12] and Endeavour [13] resulted a total of 67 genes within these regions of homozygosity. Two candidate genes (*LIG4* (MIM:*601837); *CENPT*) were identified following manual annotation and filtration by biological function (S1 Table). Simultaneous whole exome and subsequent bidirectional Sanger sequencing of both genes identified one homozygous c.1186+1G>A (GRCH37/hg19—chr16: 67863667) donor splice-site variant in exon 12 of *CENPT* on chromosome 16 in both affected siblings (IA and IC) (Fig 1B and 1C). Both parents (IIA and IIB) and the unaffected brother (IB) were heterozygous. The variant followed an autosomal-recessive inheritance pattern within the nine family members that were tested (Fig 1B) and was absent in over 600 Turkish control alleles, as well as the dbSNP database [14], NHLBI Exome Variant Server [15] and the ExAc browser [16, 17]. We excluded the possible involvement of other previously reported genes associated with dwarfism or microcephaly (the full list of genes is available in S2 Table) by targeted variant filtering and annotation in the WES data using Gem.app [18].

To test the transcriptional consequences of the homozygous donor splice-site variant, we extracted total RNA from EBV transformed lymphoblastoid cell lines (LCLs) of all three siblings and both parents. Next, unselected cDNA was synthesized using Superscript III (Thermo Scientific) and a 1:1 ratio of Oligo dT and random hexamer primers. Downstream RT-PCR using gene-specific primers amplifying exons 12–16, showed aberrant *CENPT* splicing in the affected children, their brother and parents. We verified the absence of the wild-type (WT) isoform in both affected siblings (Fig 1D), and the presence of both WT and mutated isoforms in the heterozygous parents (S1 Fig) using the Agilent DNA 7500 Bioanalyzer chip. Subsequent sub-cloning and Sanger sequencing of the gel-purified RT-PCR products confirmed the absence of the WT isoform as well as the presence of three novel alternatively spliced isoforms (r.[1030_1186del, 1024_1186del, 1022_1186del]; p.Val344Glnfs*65, p.Val342Glnfs*65, p.Gly341_Ala395del) in both affected siblings (Fig 1D). One of these three alternative isoforms (r.1022_1186del, p.Gly341_Ala395del) results in an in-frame deletion sparing the highly conserved C-terminal histone-fold domain (HFD) of CENPT, which is in accordance with a proposed isoform model published on ENSEMBL (ENST00000564817/CENPT-008; Ensembl release 87) [19]. The remaining two isoforms are predicted to cause out-of-frame deletions resulting in premature termination codons and subsequent truncation of the protein affecting the HFD domain (Fig 1E). The endogenously occurring in-frame isoform shows a higher expression than the two out-of frame isoforms in both the homozygous children and heterozygous parents, while only minimal expression is detectable in the normal control sample. It causes a 55 amino acid deletion upstream of the HFD, which is also conserved throughout placental species. This preferential expression of the in-frame isoform, which retains the HFD (Fig 1E) likely represents an intrinsic rescue mechanism.

**Fig 2:**
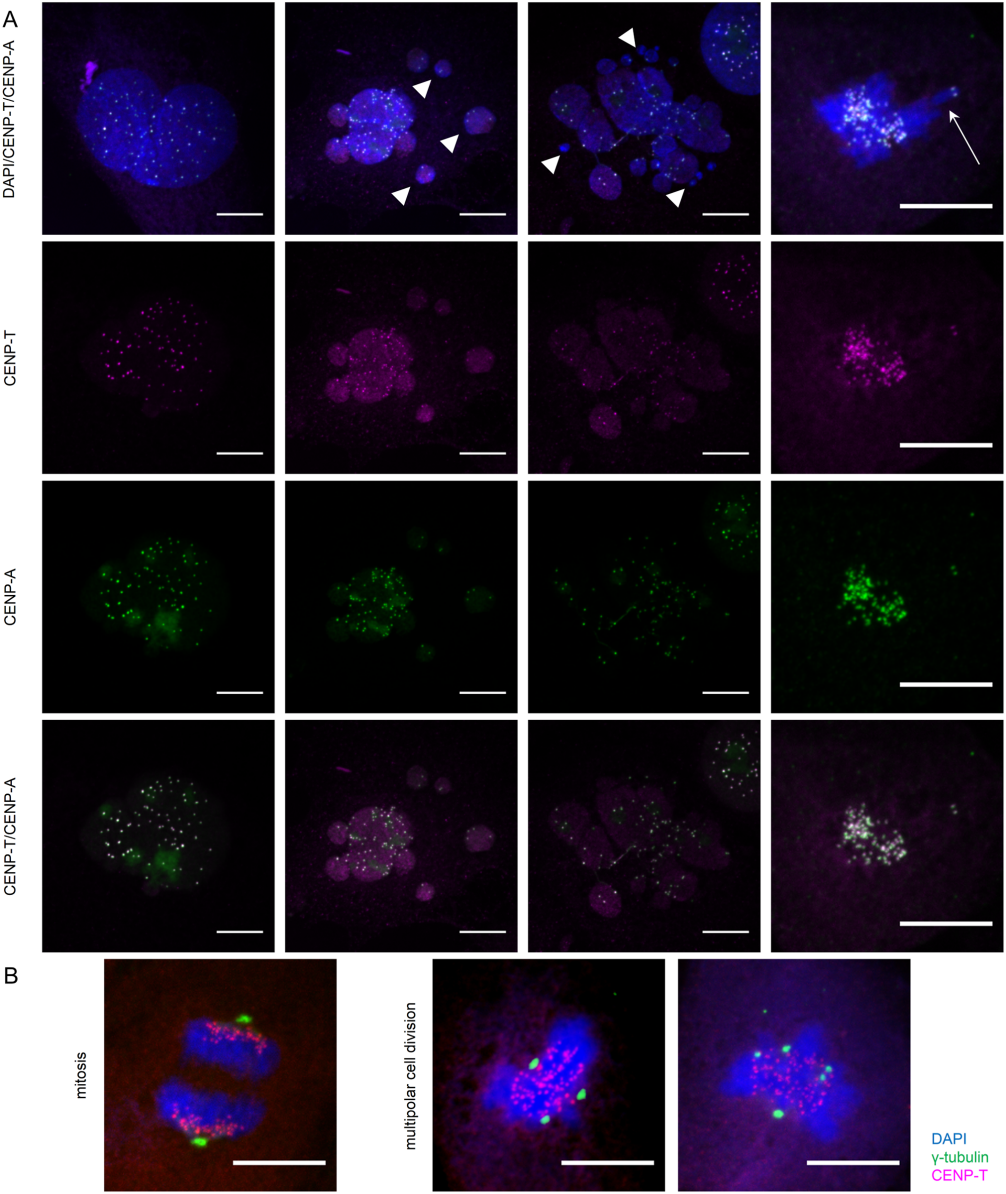
Cellular aberrations in patient fibroblast lines. (A) Immunofluorescence staining of immortalized fibroblasts of index IA; blue—DAPI; magenta—CENPT; green—CENPA or phosphorylated CENPA. Scale bars represent 10μm. Multinucleated cells are depicted in columns 1–3. Micronuclei (solid arrowheads) and chromosome lagging (arrow). (B) Normal mitosis on the left. Examples of multipolar mitosis on the right in immortalized fibroblast of index IA. Blue—DAPI; magenta—CENPT; green–γ-tubulin. Scale bars represent 10μm.

**Fig 3:**
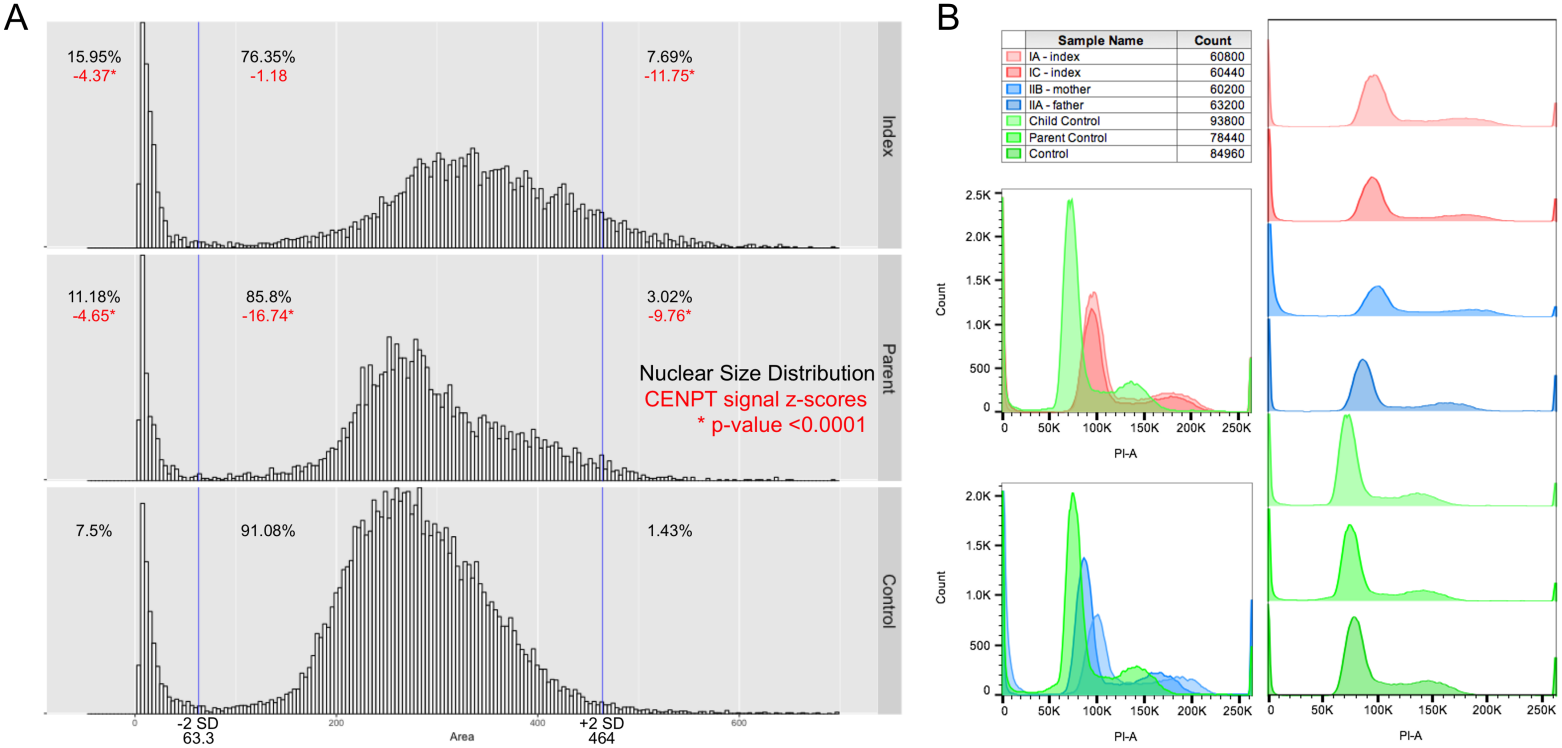
Cell size distribution. (A) Nuclear size distribution and CENPT signal intensity in immortalized fibroblasts measured by immunofluorescence. CENPT signal intensities are age-corrected. (B) Right-shifted histograms of patient (red) and parent (blue) EBV-transformed lymphoblast cell lines in relation to three different controls (green) by flow cytometry staining. Propidium iodide was used as DNA stain.

To assess the functional consequences of the human *CENPT* splice mutation, we performed immunofluorescent staining on immortalized fibroblasts of the affected girl (IA), the mother (IIB) and two female controls of different ages (child and adult) using DAPI for DNA content, antibodies against γ-tubulin for centriole localization, CENPA and pCENPA for centromeric staining and CENPT. E6/E7 transformed skin fibroblasts of the same passage (P8-P10) were plated simultaneously in 4-well glass chamber slides, double-thymidine blocked and either released for 4 hours or treated with nocodazole for 6 hours prior to fixation and staining to enrich for cells in late S- or M-phase. The CENPT antibody (MBL D286-3, clone 42F10) used for these experiments targets the N-terminus of the protein, identifying all isoforms present in the index family. First, eight series of 5-frame stitches were taken of each well stained for DAPI, γ-tubulin and CENPT using a 20x objective of a Nikon TiEclipse inverted microscope. A minimum of 5 randomly chosen series per sample was analyzed and quantified using FIJI [20]. Briefly, regions of interest (ROI) were defined and manually curated using the DAPI channel. Subsequently, the mask was used to quantify CENPT signal intensity within each nucleus and normalized to nuclear size. For further delineation of possible kinetochore localization defects, we stained the same fibroblast lines for DAPI, CENPA or phosphorylated CENPA and CENPT. We observed a tendency towards multinucleated cells (Fig 2A three left columns) and micronuclei formation (Fig 2A solid arrowheads) by immunofluorescence. However, no statistically significant increase in mitotic aberrations, such as multipolar divisions (Fig 2B), chromosome fragmentation or lagging (Fig 2A arrow), was noted. Centromeric localization patterns of CENPT were not obviously disturbed in patient cell lines as CENPT co-localized with CENPA and phosphorylated CENPA during mitosis. This result can be explained by conservation of the HFD domain in the in-frame isoform, which is preferentially expressed. Increased proportions of oversized (>464μm^2^, +2SD of control) and undersized (<63.3μm^2^, -2SD of control) nuclei were measured, which occurred to greater extent in the affected (7.69% and 15.95%) compared to the parent (3.02% and 11.18%) or the controls (1.43% and 7.5%) (Fig 3A). The quantifications and measurements therefore confirm the subjective microscopic observations of increased micronuclei formation and of cell morphology suggesting either a delay in S-phase progression or incomplete cell division. Furthermore, CENPT expression within the nuclei were significantly lower in the affected and parent when controlled for age group and stratified by nuclear size with obvious reduction of signal intensity occurring in the oversized nuclei (-11.75SD and -9.76SD, p = 0.0001), followed by the micronuclei (-4.37SD and -4.65SD, p = 0.0001) (Fig 3A). Crude counting of centromeres in observed meganuclei and multinucleated cells, indeed resulted in higher numbers (>60) of centromeres compared to regular sized cells representative of polyploid cells. As the cellular findings in the fibroblasts from the index and parent are in accordance with previously described cellular phenotypes of other primordial dwarfism syndromes [8, 21], we next tested for possible downstream effects. The extreme global growth failure phenotype is a manifestation of overall reduced cellularity of an affected organism [1]. However, it is not known whether reduced cellularity is caused by decreased cell proliferation, increased cell apoptosis or other mechanisms. To test for cell cycle aberrations and apoptosis rates, LCLs and immortalized fibroblasts were either stained with propidium iodide (PI) or with the Alexa Fluor 488 annexin V/Dead Cell Apoptosis Kit (Invitrogen) prior to analysis by flow cytometry. FlowJo V10 (FlowJo, LLC) was used for data analysis. No differences in cell cycle profiles (S2 Fig) or cell apoptosis rates were found in LCLs (Fig 3C) when comparing the two affected children, their parents and three controls. However, a higher S-phase proportion (33.2% vs 13.9%) was seen in the immortalized fibroblasts (P9) of the two affected children when compared to immortalized control fibroblasts of matched passages. No significant differences were observed in the G0/G1 and G2/M phases (for cell cycle profiles see S3 Fig). The increase of cells in the S phase is a direct consequence of increased DNA dye uptake in the oversized cell nuclei and multinucleated cells as observed by immunofluorescence. In addition, flow cytometry showed a consistent right shift of patient and parent cells compared to control in both lymphoblast (Fig 3B) and fibroblast cell lines (S4 Fig) when stained with propidium iodide, which emphasizes the differences in DNA content once again. Although these findings did not show altered mitotic or apoptotic rates, they are all in agreement with previously reported subtle defects in G1/S transition and S-phase progression in cell lines of individuals with primordial dwarfism [21]. Furthermore, CENPT recruitment is known to occur during S-phase [22] and studies have implicated that centromere integrity is maintained outside of mitosis, presumably in S-phase through CENP-A/CCAN-dependent mechanisms [23]. Future studies will be needed to determine whether these subtle S-phase changes prove to be a consistent hallmark in individuals with *CENPT* variants.

**Fig 4:**
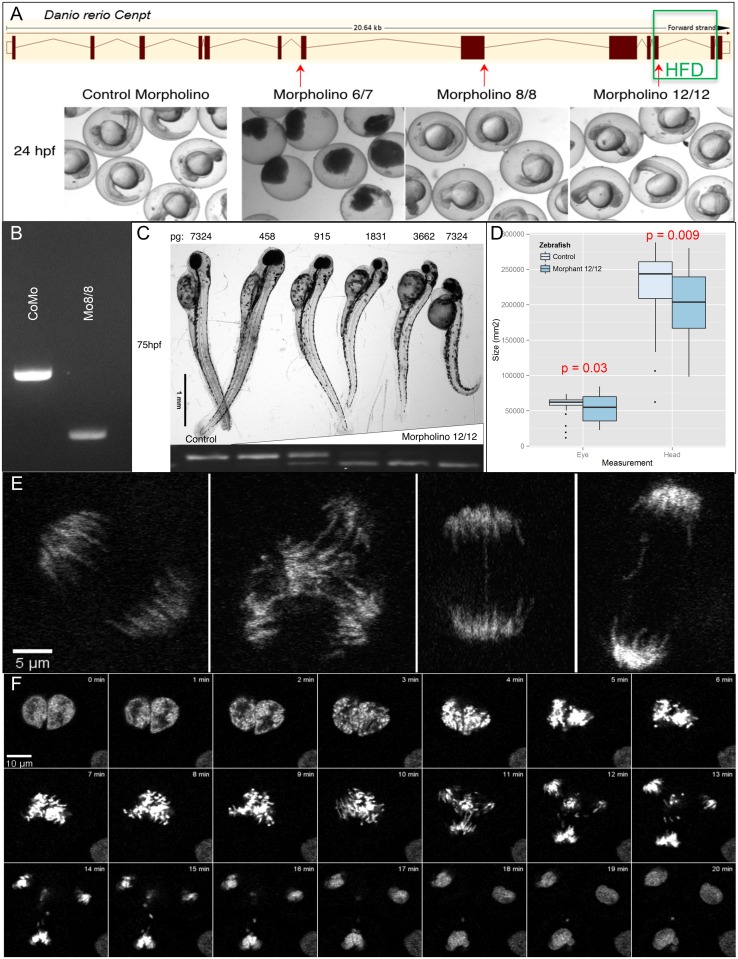
Knock-down zebrafish model. (A) Comparison of morpholino target effects at 24hpf and localization of morpholino targets in relation to the zebrafish *cenpt* gene and the conserved histone-fold domain (HFD, green box). (B) Effect of Mo8 (Mo8/8) on transcription of zebrafish *cenpt* in comparison to control morpholino (CoMo). (C) Dose response curve of Mo12 (Mo12/12) at 75hpf. On the bottom RT-PCR of Mo12 dose response curve. The upper band represents the wild-type band. (D) Head and eye measurements in Mo12 injected zebrafish at 48hpf. (E) Left panel shows a normal mitosis. The three right panels are examples of mitotic aberrations seen in Mo12 injected Tg(h2afv:GFP) zebrafish live imaging. (F) Time lapse of aberrant mitosis in Mo12 injected Tg(h2afv:GFP) zebrafish.

To mimic the effects of the splice variant in an additional in vivo system, while taking into account a possible intrinsic rescue mechanism as suggested by the RT-PCR experiments, a splice-site targeting morpholino knock-down strategy was assayed in developing zebrafish. Zebrafish injected with morpholino antisense oligos targeting different splice sites of the *Danio rerio cenpt* gene (exon-intron junctions 6/7, 8/8 and 12/12; Fig 4A) developed a spectrum of dysmorphic phenotypic features compared to control morpholino (CoMo) injected zebrafish, ranging from decreased body length and head size to death. The degree of lethality and dysmorphism depended on the proximity of the morpholino target sequence to the 5’ end of the gene, with morpholinos targeting further 5’ prime (N-terminal) causing a more severe phenotype (Fig 4A). Furthermore, dose response experiments in embryos injected with morpholino targeting the exon 12—intron 12 junction (Mo12), the furthest 3’ prime (C-terminal) targeting morpholino, resulted in a decrease in head and body size inversely proportional to the morpholino dosage at 75 hours post fertilization (hpf) (Fig 4C). At 48hpf, zebrafish injected with 576pg Mo12 (n = 46) showed a trend toward decreased body length compared to control (n = 46) fish (4.75%, p = 0.08). However, decreases in head (11%, p = 0.0091) and eye (10.3%, p = 0.0341) size were highly significant (Fig 4D). More importantly, Mo12, whose orthologous target most closely resembles the splice-site affected in the probands (Fig 4A), induced a less severe phenotype with significantly decreased body length and head size. The target specificity of both Mo8, which targets the exon 8 –intron 8 junction, and Mo12 were assessed by RT-PCR as shown in Fig 4B and 4C. At the cellular level, pre-gastrulation (3–8 hpf) live imaging of mitosis in Tg(h2afv:GFP) embryos (ZIRC) showed an increased tendency for multipolar cell divisions (n = 392 mitoses; RR 1.74; CI 1.01–3.02) and chromosome lagging or fragmentation (n = 306 mitoses; RR 2.5; CI 1.53–4.09) in Mo8 injected embryos. Mo12 injected zebrafish also exhibited increased chromosomal lagging or fragmentation (n = 280 mitoses; RR 2.23; CI 1.35–3.69) but did not result in an increase in multipolar divisions (n = 344 mitoses; RR = 0.51; CI 0.24–1.1) (Fig 4E and 4F). Therefore, the zebrafish Cenpt knock-down model, particularly Mo12, recapitulates the key aspects of the human clinical and cellular phenotypes and confirms the biological importance of Cenpt for brain and somatic growth.

To identify additional affected individuals, 59 patients with matching HPO terms (microcephaly, microcephaly and short stature, primordial dwarfism) or ICD-9 codes from various institutions (University of Miami, Vanderbilt University, University of Edinburgh) were screened for *CENPT* variants by Sanger sequencing of all *CENPT* exons and exon/intron boundaries (GenBank: NM_025082.3). Through various databases (CentoMD^®^ [24], MatchMaker Exchange [25]) and clinical collaborators, we identified 9 additional individuals in 8 families with microcephaly and growth delay carrying missense variants in *CENPT* in the homozygous or compound heterozygous state (Fig 1E pink markings). However, no detailed phenotypic information or patient-derived samples could be obtained to examine the pathogenicity of these variants.

## Discussion

In summary, we identified a homozygous splice-site mutation in *CENPT* and confirmed its aberrant splicing effect at the transcript level in two siblings with severe microcephaly and short stature born to consanguineous parents from Turkey. A Cenpt knock-down zebrafish model resembles the human phenotype and is in-line with other reported microcephaly zebrafish model findings, especially with regards to the head and eye presentations [26]. The non-significant decrease in length in the *cenpt* morphants may reflect incomplete knock-down of Cenpt, since distinct changes in body length only at dosages higher than 1831pg (Fig 3B). While the results of the zebrafish knock-down experiments match the clinical and cellular phenotypes of the patients, it can be argued that knock-down of any centromere protein (CENP) may yield similar cellular phenotypes. It is noteworthy however, that N-terminal knock-down of Cenpt and high doses of morpholino resulted in non-viable zebrafish suggesting that only incomplete knock-down is compatible with life. This is in biological agreement with the mutation found in the two affected children, as one of the resulting isoforms spares the highly conserved C-terminal HFD domain resulting in an intrinsic rescue mechanism as described to occur in other primordial dwarfism syndromes [27]. Putting the highly conserved and central role of kinetochore in cell division into perspective, it is plausible to assume that defects in these proteins are rarely viable and may even undergo negative selection. In fact, the rarity of combined *CENPT* missense (excluding alleles with rs identifiers), nonsense and splice site variants in the general population according to the ExAc database [17] (MAF 0.00076) highlights the gene’s critical role for development, suggesting our finding to be an exception to lethality, due to partial rescue through the alternatively spliced in-frame isoform in these siblings.

Sequence alignments of orthologous CENPT in different placental species [19] furthermore underline the high degree of conservation of the C-terminal HFD, which is required for correct kinetochore location to centromeres [22]. Our immunostaining data confirms that the preservation of the HFD is sufficient for correct recruitment of CENPT to the centromere. No changes of localization patterns were found, supporting that the in-frame isoform represents the main isoform in the two siblings as suggested by the RT-PCR analyses. As a part of CCAN (constitutive centromere-associated network) [28, 29], CENPT stretches under tension [30] enabling it to gauge the pulling forces responsible for faithful sister chromatid segregation during anaphase [31]. In vitro experiments have shown that the N-terminal region of CENPT is responsible for recruiting and maintaining kinetochore interaction and suggest that kinetochore formation and microtubule spindle assembly are stable in C-terminus depleted CENPT human U2OS and chicken DT40 cell lines [32]. Down-regulation of CENPT by siRNA in hTERT-RPE1 (retinal pigmented epithelial 1) caused micronuclei formation due to chromosome missegregation [23]. Studies have also reported multipolar mitoses, chromosome lagging and fragmentation with subsequent micronuclei formation as consequence of CENPT depletion in cell models and proven the importance of CENPT localization during S-phase [22, 23, 32–34]. Future key experiments to test if the mutation affects the assembly of nucleosome-like structure that CENP-T forms with CENPs-W, -S, and-X, will be needed to support our proposed pathomechanism in these patients. Although the *Danio rerio* Cenpt protein exhibits a species-specific sequence N-terminal of HFD, the knock-down model was able to replicate those aspects including multipolar cell divisions and chromosome lagging and fragmentation in live imaging. Similar cellular defects were observed in patient-derived cell lines. Even though no statistical increase in mitotic defects could be directly observed through immunofluorescence of the human fibroblasts, the increased formation of micronuclei, nuclei of increased size and multinucleated cells point to the same conclusions. Furthermore, it should be noted that multipolar divisions were only observed in the N-terminally targeted zebrafish morphants (Mo8), which concurs with the concept that the N-terminus of CENPT is responsible for recruitment and stabilization of outer kinetochore machinery. Therefore, the consequence of the described variant may not elicit as strong of an effect on the spindle apparatus assembly or kinetochore recruitment but rather influence other proposed regulatory functions of the inner kinetochore during S-phase. Taken together, these studies support a critical role of CENPT during cell division. The differences in the *CENPT* sequence between zebrafish and human are important in interpreting the knock-down experiments and suggest that there are additional features of *CENPT* that we don’t understand and could be useful to the comparisons of the zebrafish and human phenotypes.

In conclusion, the present study highlights *CENPT* as a novel disease gene and is the first to suggest the involvement of a constitutive and inner kinetochore/CCAN member [35] in the pathogenesis of severe growth deficiency. These studies emphasize the involvement of kinetochore defects as an additional patho-mechanism for these severe microcephalic growth disorders.

## Materials and methods

### Subjects

This study was approved through the Institutional Review Board at the University of Miami (#20081166). Written informed consent from the family was given for the samples (blood samples, skin biopsies) and medical images (brain MRI) obtained. A total of nine family members were recruited (2 affected, 7 unaffected). Over 300 anonymized control samples were screened to assess the allele frequency in the Turkish population. Additional testing of 59 patients with matching HPO terms (microcephaly, microcephaly and short stature, primordial dwarfism) or ICD-9 codes was conducted. Informed consents and IRB approvals were obtained through local IRBs at the collaborating centers for the screened samples.

### Cell culture

All culturing conditions occurred at 37°C and 5% CO2. Fibroblast cultures of both affected children and both parents were grown from skin biopsies under a standard collagenase II digestion protocol. Following establishment of the primary fibroblast cell lines, each line was immortalized using human papillomavirus E6/E7 virus according to standard protocols [36]. DMEM-F12 media supplemented with either 10% (for immortalized fibroblasts) or 15% (for primary fibroblasts) fetal bovine serum and 1% antibiotics (penicillin, streptomycin) was used to grow the established cell lines.

Lymphoblast cell lines (LCLs) were established from whole blood. After isolation of peripheral blood mononuclear cells (PBMCs) using either a Ficoll gradient or CPT Vacutainer tubes (BD Biosciences), B-lymphocytes were EBV transformed and maintained in RPMI-1640 media supplemented with 15% fetal bovine serum and 1% antibiotics (penicillin, streptomycin).

### Autozygosity mapping

Genomic DNA quality and quantity was evaluated via gel electrophoresis and ND-8000 spectrophotometer. DNA samples were processed according to Affymetrix procedures for Genome-Wide Human SNP Array 5.0. Briefly, DNA was digested with *NspI* and *StyI* restriction enzymes and ligated to adaptors that recognize the cohesive 4bp overhangs. The adaptor-ligated DNA fragments were amplified in a PCR reaction using a generic primer that recognized the adaptor sequence. PCR conditions were set to preferentially amplify fragments ranging from 200-1100bp. Fragment size was verified by agarose gel electrophoresis. The amplified products were pooled and purified using polystyrene beads, fragmented, labeled and loaded on the Genome-Wide Human SNP Array 5.0. Hybridization occurred overnight in a GeneChip^®^ Hybridization Oven at 50°C. Post-hybridization, the arrays were washed and stained using a Streptavidin Phycoerythrin (SAPE) staining solution on the Fluidics Station 450. The arrays were subsequently scanned on a GeneChip Scanner 3000 7G. Raw data was analyzed using the Genotyping Console^™^. Samples with call rates below 98% were excluded from analysis. Shared autozygous regions were ranked according to size and the genomic coordinates of regions of >1Mb in size were entered into ToppGene [12] and Endeavour [13] for gene prioritization. The resulting 67 candidate genes were subsequently ranked after manual curation.

### Bi-directional Sanger sequencing

Gene-specific primers were designed to include a minimum of 20bp intronic sequence from the exon-intron boundaries of each exon. PCR amplification was performed on Veriti thermal cyclers (ABI) from genomic DNA extracted from whole blood. Following PCR product clean-up using Sephadex G-50 Fine DNA grade (GE Health) columns in combination with SOPE Resin (Edge Bio), bi-directional sequencing reactions were set-up using the gene specific primers and Big Dye Terminator v3.1 (ABI) chemistry. After another Sephadex clean-up the sequencing reactions were analyzed by capillary electrophoresis on either an ABI 3130 or 3730. Sequence traces were aligned and analyzed using Mutation Surveyor (Softgenetics). Specific primer sequences and thermal cycling conditions are available upon request.

### Whole-exome sequencing

Library construction and capture hybridization was performed using the SureSelect XT Human All Exon V4 kit (Agilent Technologies) according to the manufacturer’s protocol. Samples were barcoded post-capture to allow for multiplexing of 4 samples per HiSeq2000 lane. Cluster generation took place on the Illumina cBot according to the manufacturer’s recommendations. Sequencing occurred on the Illumina HiSeq2000 using the reagents provided in the Illumina TruSeq PE Cluster Kit v3 and the TruSeq SBS Kit-HS (200 cycle) kit. An average of 81.4 million pass filter paired-end reads per sample were generated for an average depth of 45x. Filtering was performed using the software GEM.app [18].

### RT-PCR

Total RNA was extracted from whole blood and subjected to DNAse treatment prior to cDNA synthesis using SuperScript III (Thermo Scientific) and a 1:1 ratio of Oligo dT and random hexamer primers according to the manufacturer’s specification. Exon spanning gene specific primers were used to amplify the regions of interest, which spanned exon 12–16. Specific primer sequences and thermal cycling conditions are available upon request. RT-PCR products were subsequently loaded on 2% Agarose gels and run for 2h at 70V. All visible bands of each affected individual and each parent were gel extracted using the Qiagen MinElute Gel Extraction kit and sub-cloned using the Zero Blunt TOPO PCR Cloning kit (Thermo Scientific) according to the manufacturers’ protocols. A total of 50 clones per individual were randomly selected and sequenced using M13 primers. Sequence traces were aligned to the mRNA reference sequence NM_025082.3 and compared to available transcripts in both RefSeq [37] and Ensemble [19].

### Bioanalyzer

Total RNA was extracted from EBV transformed lymphocytes of both affected individuals and parents using a simultaneous DNA, RNA and protein Trizol extraction protocol [38]. RNA quality and concentrations were assed in duplicates using Agilent RNA 6000 Nano Bioanalyzer chip. All RNA samples had a RIN of 9.7 or above. Subsequently, cDNA was synthesized using Superscript III (Thermo Scientific) as previously described. Amplification was achieved using the same primers and thermal cycling conditions as in the RT-PCR experiments. Amplification was stopped during the pre-determined linear phase (31–35 cycles) to enable detection of possible differences in expression between the two affected siblings or isoforms. Finally, all PCR products were analyzed on an Agilent DNA 7500 Bioanalyzer chip. DNA from the same extraction was used to confirm the splice-site mutation in the corresponding DNA.

### Immunofluorescence

Immortalized fibroblasts of the same passages (P8-P10) from the affected girl (IA), mother (IIB) and two control females (child, adult) were plated simultaneously onto Poly-D-Lysine (50μg/ml) coated, 4-well glass chamber slides (Nunc) overnight. At 30% confluency, the cells were double-thymidine blocked (2mM thymidine for 19hrs each with 6hrs release in between). Following either a 6hrs nocodazole (40ng/μl) treatment or a final 4hrs release, cells were fixed in 4% paraformaldehyde for 15min at room temperature, washed in phosphate buffered saline, then permeabilized and blocked (10% normal donkey serum, 0.1% Triton X-100 in PBS) for 30min. Slides were incubated in primary antibodies for 2hrs at RT or overnight at 4°C, washed, incubated in secondary antibodies for 1hr at room temperature and mounted with ProLong Gold anti-fade media with DAPI (Thermo Scientific). Monoclonal rat anti-CENPT (MBL D286-3, clone 42F10), monoclonal rabbit anti-γ-Tubulin (abcam ab179503, clone EPR16793), polyclonal rabbit anti-CENPA (Cell Signaling Technology 2186) and polyclonal rabbit anti-phospho-CENPA (Cell Signaling Technology 2187, Ser7) primary antibodies were used at manufacturer recommended concentrations. Secondary antibodies were affinity-purified anti-IgG antibodies from Jackson ImmunoResearch Laboratories Inc.

For quantification, eight series of 5-frame stitches were taken of each well using the 20x objective of a Nikon Ti Eclipse inverted microscope. A minimum of 5 randomly chosen series per sample was analyzed and quantified using FIJI [20]. In short, nuclei were identified as regions of interest (ROI) in the DAPI channel using a macro. Subsequently all ROI were manually curated to identify falsely registered or missed ROI. After curation the ROI were used as mask to measure area and signal intensities within the ROI on the DAPI and CENPT channels. Each series was systematically screened for aberrant mitoses and the polarity of mitoses was determined by the γ-tubulin signal distribution in mitotic cells. R [39] was used for statistical calculations.

For CENPT localization assessments, a Perkin Elmer Ultraview Vox spinning disk confocal microscope was used in combination with a 60x lens. Z-stacks with 2μm Z-steps were recorded to ensure complete capture of all kinetochores within the nuclear thickness. FIJI [20] was used to obtain Z-projections (maximum or standard deviation) for assessment of localization patterns.

### Zebrafish model

A knockdown zebrafish model was established using in-vivo injections of morpholinos into fertilized zebrafish eggs prior to the 2-cell stage. Morpholinos were designed to modify splicing prior to or in the conserved histone homology region in order to resemble the human molecular variant as closely as possible. A dose-response curve was established for all morpholinos. Modified splicing following morpholino injection was confirmed by RT-PCR for Mo8 and Mo12 (Fig 4B and 4C). Zebrafish morphology was assessed by brightfield microscopy at different time points.

#### Zebrafish measurements

A thin 2% agarose gel with 1mm wells was poured using agarose gel combs. The solidified gel was then cut horizontally to remove the bottom of each gel well. Subsequently the gel was flipped and cut to fit into a 15 cm petri dish. Zebrafish were anaesthetized with 3-aminobenzoic acid ethyl ester (765μM, MS222, Sigma-Aldrich) and one fish was placed in each well. Each fish was aligned against the bottom of the well/petri dish and turned to lie on its side. Pictures were taken of each fish using the same objective (20x) and setting for each injection group. All measurements were obtained using FIJI software [20]. Prior to measuring the fish, the thickness of each well was measured three times (at both ends and in the middle) and averaged within each injection group. The average pixel of each group was used to set the scale of 1mm (CV was well below 10%) and applied to all measurements within that injection group. Measurements were conducted using the ROI functions in FIJI.

#### Zebrafish live imaging

Confocal whole-mount live cell imaging between 3–8 hpf was conducted after morpholino injection of transgenic Tg(h2afv:GFP) fish (ZIRC) to visualize mitosis. First, female Tg(h2afv:GFP) were crossed with WT males. Injections were only performed on fertilized eggs at the 1-cell stage with boluses placed into the yolk as close to the cell as possible. Bolus size was controlled for using a micrometer. Eggs from the same batch were used for the injections of the morpholinos and their corresponding control to avoid any differences in genetic and environmental background. Injected embryos were kept at 27°C until mounting for imaging but sorted for viability, bolus dye distribution and fluorescence at 1–2 hpf. To mount the embryos for live imaging, 1% agarose was poured into a glass bottom dish. After solidification, holes were punched using a glass pasteur pipette. The dish was subsequently filled with embryo raising media. Embryos were removed from the incubator and de-chorionated using 1mg/ml pronase for approximately 6 minutes. Following de-chorionization, embryos were transferred individually into the punched agarose wells (1 per well) using a glass pasteur pipette. Once the embryos were placed into their wells, as much embryo raising media was removed as possible and the wells were overlaid with 3% methylcellulose. After 15–30 min, embryos were oriented using a hair loop and imaged for approximately 1 hour each on a Leica SP5 confocal microscope. All image analyses including counting and tracking of mitoses were done using FIJI [20].

### Flow cytometry

All flow cytometry experiments were run on a LSR Fortessa (BD). All analyses were performed using FlowJo V10 (FlowJo, LLC). For cell cycle analyses, immortalized fibroblasts and LCLs were harvested after an overnight incubation in fresh growth media and fixed in 70% ethanol for 2hrs at room temperature. Fixed cells were subsequently stained in a buffer containing propidium iodide (PI), Triton X-100 and RNase A for 30min at room temperature. To test for apoptosis in LCLs, we used the Alexa Fluor 488 annexin V/Dead Cell Apoptosis Kit (Invitrogen) according to the manufacturer’s protocol.

## Web resources

Database of Single Nucleotide Polymorphisms (dbSNP). http://www.ncbi.nlm.nih.gov/SNP/

Exome Variant Server. http://evs.gs.washington.edu/EVS/

Gem.app. https://genomics.med.miami.edu/gem-app/

Height and Head circumference charts—Primary Microcephaly and Primordial Dwarfism. http://ajackson.hgu.mrc.ac.uk/wp-content/uploads/2012/07/charts.pdf

CentoMD 3.2. http://www.centomd.com

R. http://www.R-project.org

## Supporting information

S1 Table Candidate gene list based on autozygosity regions. (PDF)

S2 Table Primary microcephaly / primordial dwarfism genes screened by WES. (PDF)

S3 Table Summary of additional individuals identified through database searches. (PDF)

S1 Fig Agilent DNA 7500 Bioanalyzer results for RT-PCR. (A) In contrast to their two affected children (Fig 1D), the heterozygous parents both show wild-type and alternative isoforms in RT-PCR analysis. The normal control sample only shows minimal expression of the endogenously present in-frame splice isoform. (B) RT-PCR Bioanalyzer analysis represented as electrophoresis picture. The highest band represents the wild-type isoform.
(PDF)

S2 Fig Cell cycle flow cytometry for EBV transformed lymphoblasts. Cell cycle analysis on EBV transformed LCLs of both index patients (upper panels), parents (middle panel) and age-matched controls (lower panels). No obvious difference in cell cycle progression were detected.
(PDF)

S3 Fig Cell cycle flow cytometry for fibroblasts. Cell cycle analysis on immortalized fibroblasts of both index patients (upper panels) and matched passages of an age-matched control (lower panels). A higher S-phase proportion (33.2% vs 13.9%) was seen in the two affected children. No significant differences were observed in the G0/G1 and G2/M phase.
(PDF)

S4 Fig Differences in DNA content in immortalized fibroblasts. Right shifted histograms in both index patients (red) and parents (blue) in comparison to cell lines matched for age and passage.
(PDF)

S1 Video Live imaging of Tg(h2afv:GFP) zebrafish embryos at pre-gastrulation. Mitotic aberrations in Mo12 injected transgenic zebrafish expressing GFP tagged histone 2A include multipolar divisions, chromosome lagging and fragmentation, and micronuclei formation.
(AVI)
