# Supplementary material for: A defect in the inner kinetochore protein CENPT causes a new syndrome of severe growth failure
Source: PLoS One. 2017 Dec 11;12(12):e0189324. doi: 10.1371/journal.pone.0189324 (PMC5724856; doi:10.1371/journal.pone.0189324)
Supplement: S1 Table — (PDF) [file pone.0189324.s001.pdf]

S1 Table. Candidate gene list based on autozygosity regions.

ToppGene	Endeavour
chr16: 57,159,402-72,236,679 (15.08Mb)	
E2F4	NUTF2
TPPP3	COG4
PSKH1	DHX38
NOL3	VAC14
**CENPT**	PSMB10
TK2	CNOT1
PARD6A	SF3B3
CKLF	C16ORF48
CYB5B	KIAA0174
ACD	NFAT5
chr5: 154,526,927-161,668,337 (7.14Mb)	
CLINT1	CLINT1
EBF1	ATP10B
C1QTNF2	TTC1
LSM11	IL12B
TTC1	EBF1
PPP1R2P3	ADRA1B
THG1L	GABRA1
ADRA1B	ITK
MED7	GABRG2
FABP6	CYFIP2

ToppGene	Endeavour
chr10: 7,036,894-12,965,875 (5.93Mb)	
CAMK1D	UPF2
UPF2	ATP5C1
TAF3	DHTKD1
DHTKD1	GATA3
USP6NL	USP6NL
SEC61A2	ECHDC3
KIN	CUGBP2
GATA3	KIN
ATP5C1	CDC123
CDC123	C10orf47
chr11: 15,717,287-18,737,952 (3.02Mb)	
GTF2H1	LDHA
TSG101	LDHC
PLEKHA7	C11ORF58
LOC494141	TSG101
RPS13	MYOD1
ABCC8	PIK3C2A
MYOD1	RPS13
LDHA	HPS5
SOX6	GTF2H1
OTOG	SOX6
chr13: 106,389,776-107,820,389 (1.43Mb)	
**LIG4**	**LIG4**
TNFSF13B	ABHD13
FAM155A	TNFSF13B
ABHD13	
