# Supplementary material for: A defect in the inner kinetochore protein CENPT causes a new syndrome of severe growth failure
Source: PLoS One. 2017 Dec 11;12(12):e0189324. doi: 10.1371/journal.pone.0189324 (PMC5724856; doi:10.1371/journal.pone.0189324)
Supplement: S2 Table — (PDF) [file pone.0189324.s002.pdf]

S2 Table. Primary microcephly / primordial dwarfism genes screened by WES.

OMIM Search - "primary microcephaly"<br>Downloaded:	Mar 30, 2017	
**Gene Name**	**HGNC symbol**	**MIM number**
CATENIN, BETA-1	CTNNB1	*116806
CENTROMERIC PROTEIN E	CENPE	*117143
CYTOCHROME C OXIDASE, SUBUNIT 8A	COX8A	*123870
INSULIN-LIKE GROWTH FACTOR I RECEPTOR	IGF1R	*147370
SCL/TAL1-INTERRUPTING LOCUS	STIL	*181590
SRY-BOX 2	SOX2	*184429
X-RAY REPAIR, COMPLEMENTING DEFECTIVE, IN CHINESE HAMSTER, 4	XRCC4	*194363
CENTROMERIC PROTEIN I	CENPI	*300065
POLYGLUTAMINE-BINDING PROTEIN 1	PQBP1	*300463
CENTROMERIC PROTEIN F	CENPF	*600236
SONIC HEDGEHOG	SHH	*600725
DUAL-SPECIFICITY TYROSINE PHOSPHORYLATION-REGULATED KINASE 1A	DYRK1A	*600855
ATR GENE	ATR	*601215
PATCHED, DROSOPHILA, HOMOLOG OF, 1	PTCH1	*601309
POLYHOMEOTIC-LIKE 1	PHC1	*602978
CYCLIN-DEPENDENT KINASE 6	CDK6	*603368
POLO-LIKE KINASE 4	PLK4	*605031
ABNORMAL SPINDLE-LIKE, MICROCEPHALY-ASSOCIATED	ASPM	*605481
CITRON RHO-INTERACTING SERINE/THREONIN KINASE	CIT	*605629
ZINC FINGER E BOX-BINDING HOMEOBOX 2	ZEB2	*605802
STAM-BINDING PROTEIN	STAMBP	*606247
MCPH1 GENE	MCPH1	*607117
CDK5 REGULATORY SUBUNIT-ASSOCIATED PROTEIN 2	CDK5RAP2	*608201
CANCER SUSCEPTIBILITY CANDIDATE 5	CASC5	*609173
CENTROMERIC PROTEIN J	CENPJ	*609279
SAS6, C. ELEGANS, HOMOLOG OF	SASS6	*609321
ROTATIN	RTTN	*610436
ZINC FINGER PROTEIN 335	ZNF335	*610827
NONHOMOLOGOUS END-JOINING FACTOR 1	NHEJ1	*611290
CENTROSOMAL PROTEIN, 135-KD	CEP135	*611423
TUBULIN, BETA-2B	TUBB2B	*612850
CENTROSOMAL PROTEIN, 152-KD	CEP152	*613529
WD REPEAT-CONTAINING PROTEIN 62	WDR62	*613583

MAJOR FACILITATOR SUPERFAMILY DOMAIN-CONTAINING PROTEIN 2A	MFSD2A	*614397
CENTROSOMAL PROTEIN, 63-KD	CEP63	*614724
tRNA METHYLTRANSFERASE 10, S. CEREVISIAE, HOMOLOG OF, A	TRMT10A	*616013
ANKYRIN REPEAT- AND LEM DOMAIN-CONTAINING PROTEIN 2	ANKLE2	*616062

**OMIM Search - dwarfism OR dwarf OR "growth retardation" OR hyposomia OR "short stature"**		
**Downloaded:**	**Mar 30, 2017**	
**Gene Name**	**HGNC symbol**	**MIM number**
NATRUIRETIC PEPTIDE RECEPTOR 2	NPR2	*108961
FIBROBLAST GROWTH FACTOR RECEPTOR 3	FGFR3	*134934
GROWTH HORMONE-RELEASING HORMONE RECEPTOR	GHRHR	*139191
GROWTH HORMONE 1	GH1	*139250
GNAS COMPLEX LOCUS	GNAS	*139320
INSULIN-LIKE GROWTH FACTOR I RECEPTOR	IGF1R	*147370
INSULIN RECEPTOR	INSR	*147670
AGGRECAN	ACAN	*155760
MITOCHONDRIAL RNA-PROCESSING ENDORIBONUCLEASE, RNA COMPONENT OF	RMRP	*157660
POU DOMAIN, CLASS 1, TRANSCRIPTION FACTOR 1	POU1F1	*173110
THYROID HORMONE RECEPTOR, BETA	THRB	*190160
X-RAY REPARI, COMPLEMENTING DEFECTIVE, IN CHINESE HAMSTER, 4	XRCC4	*194363
SHORT STATURE HOMEOBOX	SHOX	*312865
SHORT STATURE HOMEOBOX, Y-LINKED	SHOXY	*400020
HIGH MOBILITY GROUP AT-HOOK 2	HMGA2	*600698
ATR GENE	ATR	*601215
RNA, U4ATAC SMALL NUCLEAR	RNU4ATAC	*601428
PROP PAIRED-LIKE HOMEOBOX 1	PROP1	*601538
PROTEIN KINASE, cGMP-DEPENDENT, TYPE II	PRKG2	*601591
ORIGIN RECOGNITION COMPLEX, SUBUNIT 1, S. CEREVISIAE, HOMOLOG OF	ORC1	*601902
SHORT STATURE HOMEOBOX 2	SHOX2	*602504
WW DOMAIN-CONTAINING OXIDOREDUCTASE	WWOX	*605131
PERICENTRIN	PCNT	*605925
ROTATIN	RTTN	*610436
INOSITOL MONOPHOSPHATASE DOMAIN-CONTAINING PROTEIN 1	IMPAD1	*614010
POC1 CENTRIOLAR PROTEIN, CHLAMYDOMONAS, HOMOLOG OF, A	POC1A	*614783
DWARF OPEN READING FRAME	DWORF	*616891

Literature derived genes		
Version	Mar 30, 2017	
Gene Name	HGNC symbol	MIM number
CENTROMERIC PROTEIN E	CENPE	*117143
INSULIN-LIKE GROWTH FACTOR I	IGF1	*147440
MICROTUBULE-ASSOCIATED PROTEIN 4	MAP4	*157132
SCL/TAL1-INTERRUPTING LOCUS	STIL	*181590
X-RAY REPAIR, COMPLEMENTING DEFECTIVE, IN CHINESE HAMSTER, 4	XRCC4	*194363
DOUBLECORTIN	DCX	*300121
CENTROMERIC PROTEIN F	CENPF	*600236
REELIN	RELN	*600514
ATR GENE	ATR	*601215
PLATELET-ACTIVATING FACTOR ACETYLHYDROLASE, ISOFORM 1B, ALPHA SUBUNIT	PAFAH1B1 (LIS1)	*601545
LIGASE IV, DNA, ATP-DEPENDENT	LIG4	*601837
ORIGIN RECOGNITION COMPLEX, SUBUNIT 1, S. CEREVISIAE, HOMOLOG OF	ORC1	*601902
TUBULIN, ALPHA-1A	TUBA1A	*602529
CELL DIVISION CYCLE 6, S. CEREVISIAE, HOMOLOG OF	CDC6	*602627
POLYHOMEOTIC-LIKE 1	PHC1	*602978
ORIGIN RECOGNITION COMPLEX, SUBUNIT 4, S. CEREVISIAE, HOMOLOG OF	ORC4	*603056
CYCLIN-DEPENDENT KINASE 6	CDK6	*603368
RETINOBLASTOMA-BINDING PROTEIN 8	RBBP8	*604124
ABNORMAL SPINDLE-LIKE, MICROCEPHALY-ASSOCIATED	ASPM	*605481
CHROMATIN LICENSING AND DNA REPLICATION FACTOR 1	CDT1	*605525
PERICENTRIN	PCNT	*605925
MCPH1 GENE	MCPH1	*607117
ORIGIN RECOGNITION COMPLEX, SUBUNIT 6, S. CEREVISIAE, HOMOLOG OF	ORC6	*607213
CDK5 REGULATORY SUBUNIT-ASSOCIATED PROTEIN 2	CDK5RAP2	*608201
CANCER SUSCEPTIBILITY CANDIDATE 5	CASC5	*609173
CENTROMERIC PROTEIN J	CENPJ	*609279
NUDE, A. NIDULANS, HOMOLOG OF, 1	NDE1	*609449
ZINC FINGER PROTEIN 335	ZNF335	*610827
CENTROSOMAL PROTEIN, 135-KD	CEP135	*611423
CENTROSOMAL PROTEIN, 152-KD	CEP152	*613529
WD REPEAT-CONTAINING PROTEIN 62	WDR62	*613583
CENTROSOMAL PROTEIN, 63-KD	CEP63	*614724
POC1 CENTRIOLAR PROTEIN, CHLAMYDOMONAS, HOMOLOG OF, A	POC1A	*614783
