# Supplementary material for: A defect in the inner kinetochore protein CENPT causes a new syndrome of severe growth failure
Source: PLoS One. 2017 Dec 11;12(12):e0189324. doi: 10.1371/journal.pone.0189324 (PMC5724856; doi:10.1371/journal.pone.0189324)
Supplement: S3 Table — (PDF) [file pone.0189324.s003.pdf]

S3 Table. Summary of additional individuals identified through database searches.

**Subject**	**1A**	**1B**	**2A**	**3A**	**4A**	**5A**	**6A**	**7A**	**8A**
**Age**	15y		5y	37y	20.2y	5,2y	7m	9m	2,5 y
**Age at onset**			3y	2y	1,5y	4m	birth	birth	birth
**Country**	Qatar	Qatar	Saudi Arabia	Russia	Libya	Turkey	Chile	India	Oman
**Gender**	F	F	M	F	M	F	F	F	M
**Consanguinity**	Yes	Yes							
**Family history**	positive (sister)	positive (sister)	positive (sister)	negative	negative	negative	unknown	negative	negative
**Genotype**									
Allele 1	c.1562G>A	c.1562G>A	c.1334G>T	c.445G>T	c.344C>A	c.74C>T	c.213A>C	c.319T>G	c.337C>A
	p.R521Q	p.R521Q	p.R445L	p.A149S	p.P115Q	p.P25L	p.R71S	p.S107A	p.P113T
Allele 2	c.404C>T, c.523+4A>T	c.404C>T, c.523+4A>T	c.1334G>T	c.445G>T	c.344C>A	c.74C>T	c.214T>C	c.1321C>G	c.362C>A
	p. P135L, IVS8+4A>T	p. P135L, IVS8+4A>T	p.R445L	p.A149S	p.P115Q	p.P25L	p.S72P	p.P441A	p.S121Y
**Phenotype**		affected sister of 1A, no details							
Microcephaly	x		x	x	x	x	x	x	x
Growth delay	x		x	x	x	x		x	x
Dysmorphic features	x								
Micrognathia			x	x	x	x		x	x
Eye findings	Peters anomaly		ocular apraxy	normal		nd		normal	ocular abnormality, no details
Retinoblastoma			x			x		x	
Rod cone dystrophy			x	x	x	x		x	
Retinitis pigmentosa									x
Nystagmus			x	x	x	x		x	
Intellectual disability			x	x	x	x	x	x	x
Autism			x	x	x	x			
Neurologic findings									
Leukodystrophy				x					x
Seizures			x	x	x	x			x
Spasticity			x	x	x	x	x	x	x
Gait disturbance				x	x	x	x	x	
Chorea			x	x	x			x	x
Motor delay			x	x	x	x		x	x
Hypoplasia of the corpus callosum			x	x		x		x	x
Other									
Hypoparathyroidism			x			x			x
X-Ray			nd	nd		normal (skull)		normal (hand)	normal (thorax)
