# Supplementary material for: A defect in the inner kinetochore protein CENPT causes a new syndrome of severe growth failure
Source: PLoS One. 2017 Dec 11;12(12):e0189324. doi: 10.1371/journal.pone.0189324 (PMC5724856; doi:10.1371/journal.pone.0189324)

S1 Fig. Agilent DNA 7500 Bioanalyzer results for RT-PCR. (A) In contrast to their two affected children (Fig 1D), the heterozygous parents both show wild-type and alternative isoforms in RT-PCR analysis. The normal control sample only shows minimal expression of the endogenously present in-frame splice isoform. (B) RT-PCR Bioanalyzer analysis represented as electrophoresis picture. The highest band represents the wild-type isoform.

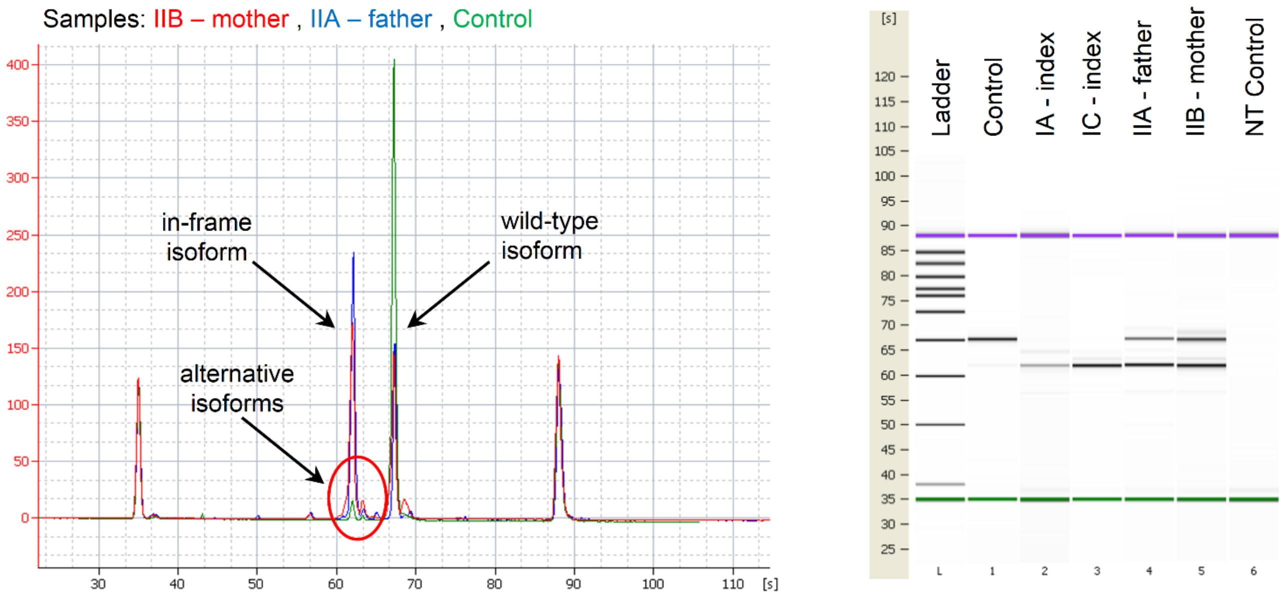

Supplement: S1 Fig — (A) In contrast to their two affected children (Fig 1D), the heterozygous parents both show wild-type and alternative isoforms in RT-PCR analysis. The normal control sample only shows minimal expression of the endogenously present in-frame splice isoform. (B) RT-PCR Bioanalyzer analysis represented as electrophoresis picture. The highest band represents the wild-type isoform. (PDF) [file pone.0189324.s004.pdf]
