# Supplementary material for: A defect in the inner kinetochore protein CENPT causes a new syndrome of severe growth failure
Source: PLoS One. 2017 Dec 11;12(12):e0189324. doi: 10.1371/journal.pone.0189324 (PMC5724856; doi:10.1371/journal.pone.0189324)

S2 Fig. Cell cycle flow cytometry for EBV transformed lymphoblasts. Cell cycle analysis on EBV transformed LCLs of both index patients (upper panels), parents (middle panel) and age-matched controls (lower panels). No obvious difference in cell cycle progression were detected.

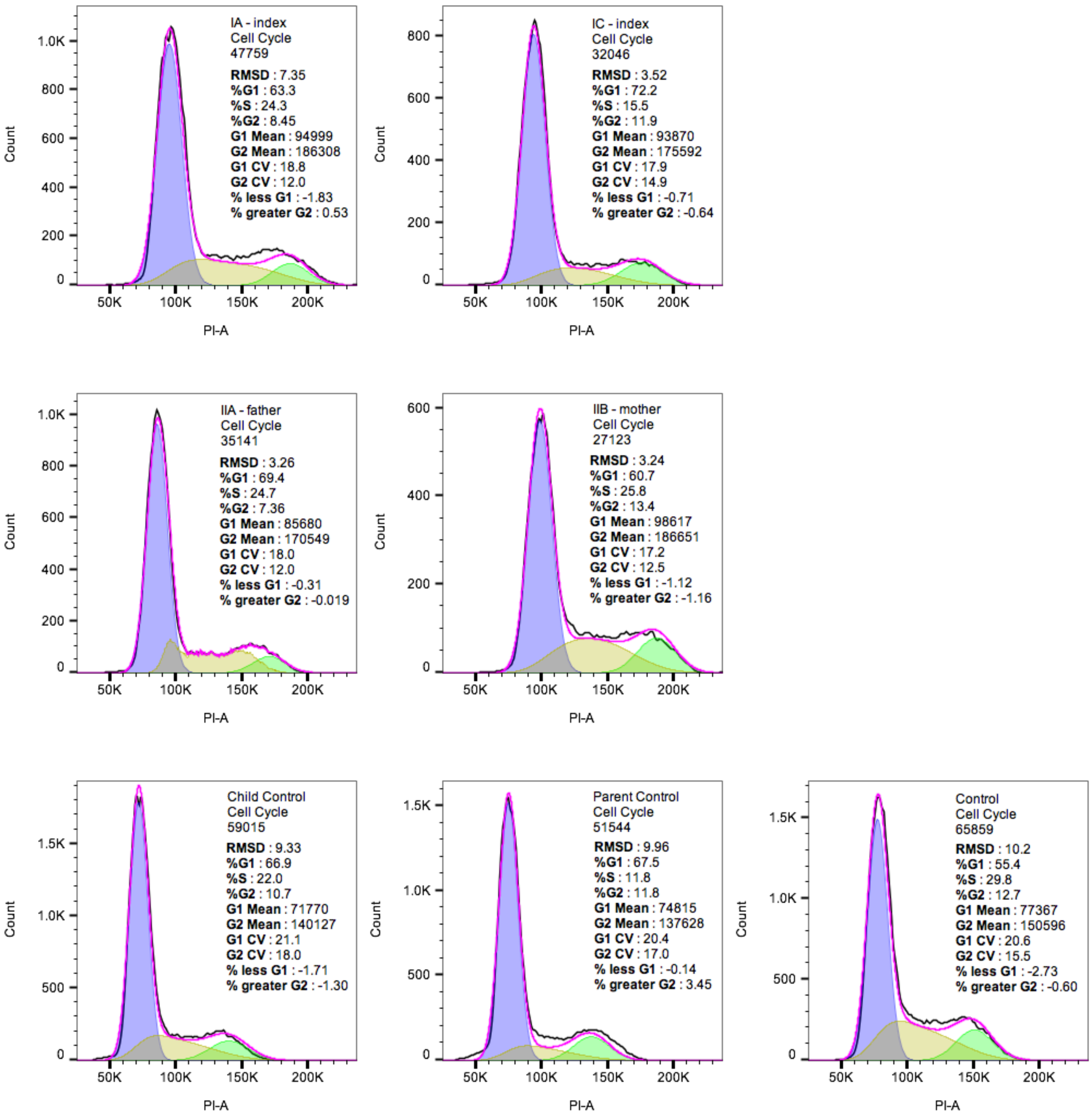

Supplement: S2 Fig — Cell cycle analysis on EBV transformed LCLs of both index patients (upper panels), parents (middle panel) and age-matched controls (lower panels). No obvious difference in cell cycle progression were detected. (PDF) [file pone.0189324.s005.pdf]
