# Supplementary material for: A defect in the inner kinetochore protein CENPT causes a new syndrome of severe growth failure
Source: PLoS One. 2017 Dec 11;12(12):e0189324. doi: 10.1371/journal.pone.0189324 (PMC5724856; doi:10.1371/journal.pone.0189324)

S3 Fig. Cell cycle flow cytometry for fibroblasts. Cell cycle analysis on immortalized fibroblasts of both index patients (upper panels) and matched passaged of an age-matched control (lower panels). A higher S-phase proportion (33.2% vs 13.9%) was seen in the two affected children. No significant differences were observed in the G0/G1 and G2/M phase.

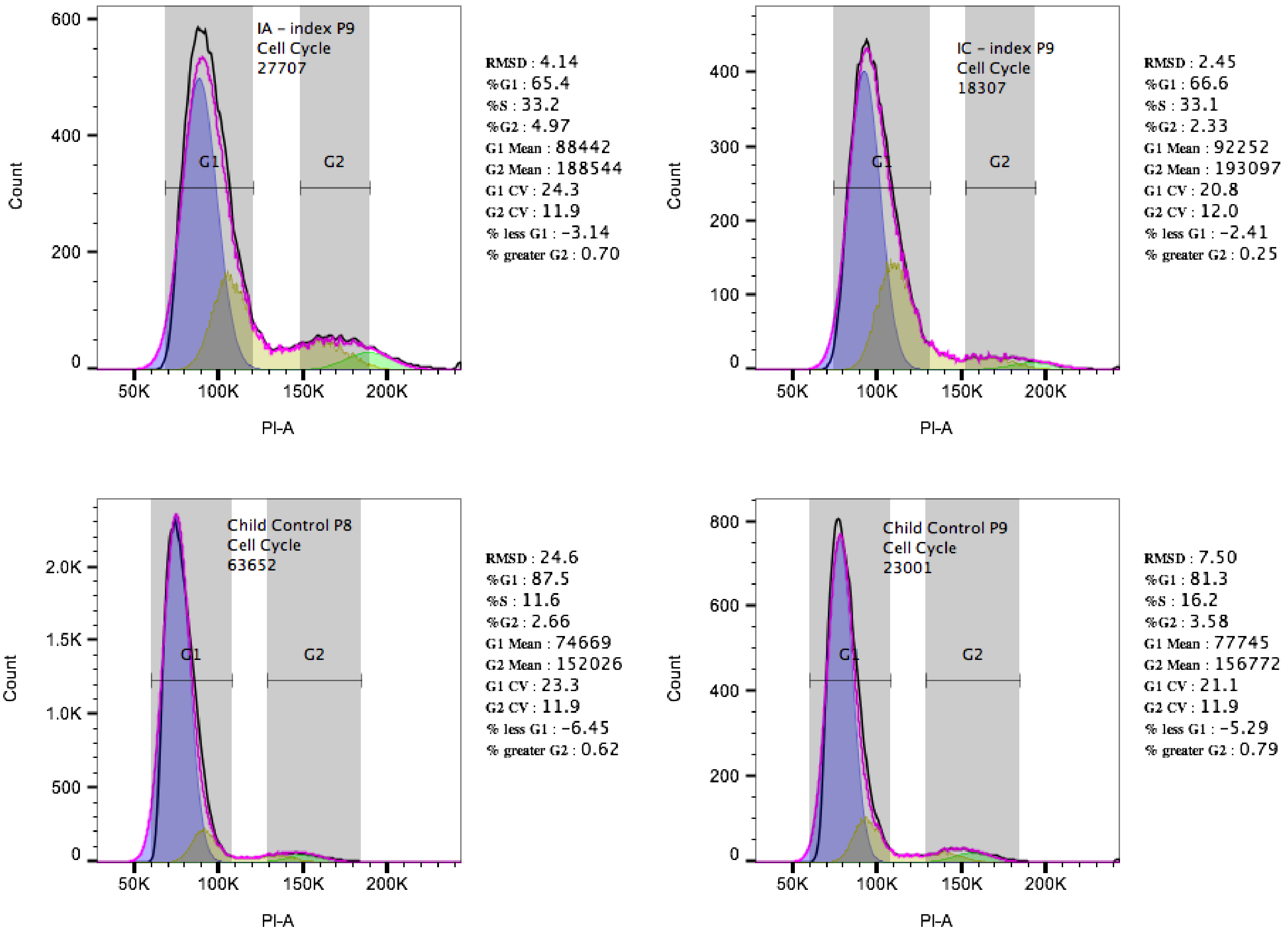

Supplement: S3 Fig — Cell cycle analysis on immortalized fibroblasts of both index patients (upper panels) and matched passages of an age-matched control (lower panels). A higher S-phase proportion (33.2% vs 13.9%) was seen in the two affected children. No significant differences were observed in the G0/G1 and G2/M phase. (PDF) [file pone.0189324.s006.pdf]
