# Supplementary material for: A defect in the inner kinetochore protein CENPT causes a new syndrome of severe growth failure
Source: PLoS One. 2017 Dec 11;12(12):e0189324. doi: 10.1371/journal.pone.0189324 (PMC5724856; doi:10.1371/journal.pone.0189324)

S4 Fig. Differences in DNA content in immortalized fibroblasts. Right shift in histograms seen in both index patients (red) and parents (blue) in comparison to cell lines matched for age and passage.

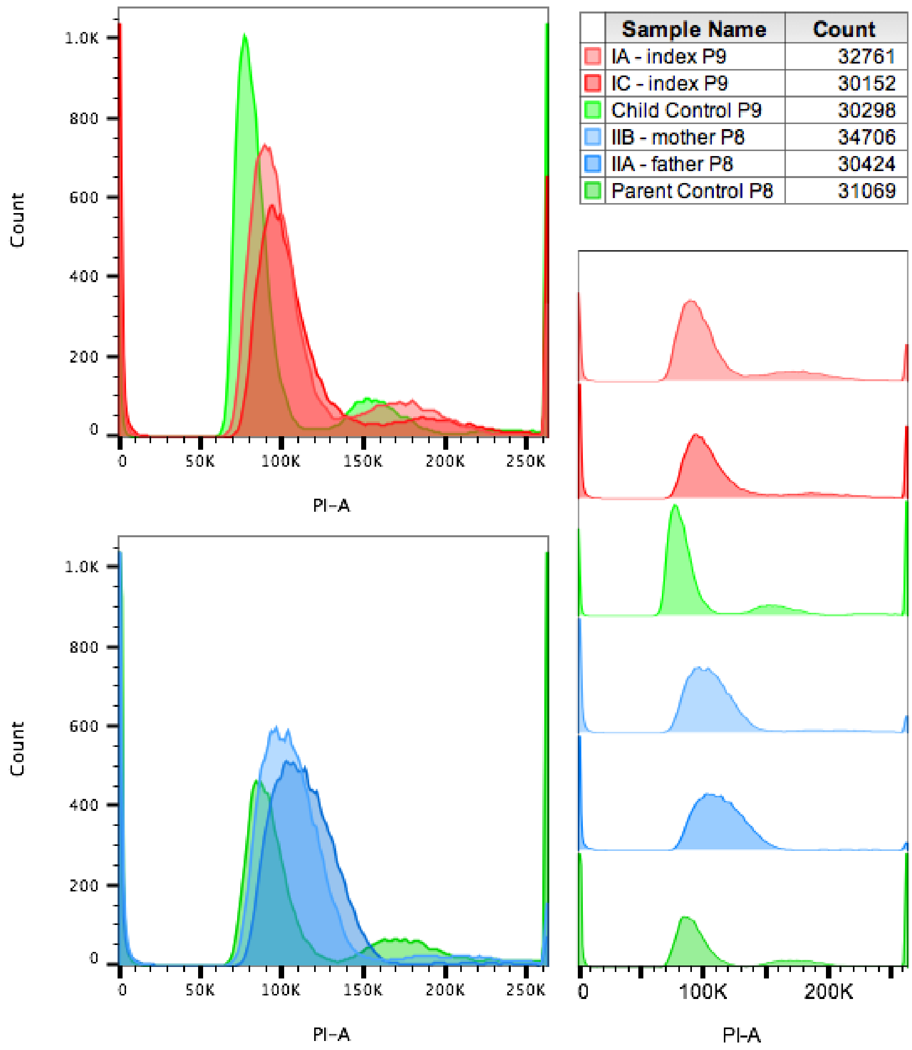

Supplement: S4 Fig — Right shifted histograms in both index patients (red) and parents (blue) in comparison to cell lines matched for age and passage. (PDF) [file pone.0189324.s007.pdf]
